# Effect of recombinant human nerve growth factor treatment on corneal nerve regeneration in patients with neurotrophic keratopathy

**DOI:** 10.3389/fnins.2023.1210179

**Published:** 2023-10-30

**Authors:** Ana Balbuena-Pareja, Chloe S. Bogen, Stephanie M. Cox, Pedram Hamrah

**Affiliations:** ^1^Center for Translational Ocular Immunology, Department of Ophthalmology, Tufts Medical Center, Tufts University School of Medicine, Boston, MA, United States; ^2^Cornea Service, New England Eye Center, Tufts Medical Center, Boston, MA, United States

**Keywords:** neurotrophic keratitis, neurotrophic keratopathy, cenegermin, recombinant human nerve growth factor, corneal nerves

## Abstract

**Introduction:**

Neurotrophic Keratopathy (NK) is a neurodegenerative corneal disease that results in diminished corneal sensation. Previous studies have found that Cenegermin 0.002%, a recombinant human nerve growth factor (rhNGF), improves corneal epithelial healing in stage 2 and 3 NK patients. However, rhNGF effect on corneal sensation and nerve regeneration has not been well established. Thus, this study aims to analyze the effect of rhNGF on corneal nerve regeneration using *in vivo* confocal microscopy (IVCM) and on corneal sensitivity in NK patients.

**Methods:**

This is a retrospective, longitudinal, case–control study that included patients with NK, treated with rhNGF for at least 4 weeks, with pre- and post-treatment IVCM images available for analysis. Chart reviews were conducted documenting prior medical and surgical history, clinical signs and symptoms, and corneal sensation using Cochet-Bonnet esthesiometry. Corneal nerve parameters were assessed by IVCM. Sex- and age-matched reference controls were selected from a database of healthy subjects for comparison.

**Results:**

The study included 25 patients, with 22 (88%) stage 1, two (8%) stage 2, and 1 (4%) stage 3 NK patients, with a median age of 64 years (range: 30–93 years). Total, main, and branch nerve densities [median (range) in mm/mm^2^] were lower in the NK group pre-treatment [2.3 (0.0–21.1); 1.7 (0.0–13.0); 0.5 (0.0–10.2); respectively] vs. controls [22.3 (14.9–29.0); 10.1 (3.2–15.4); and 12.1 (6.2–18.4), (*p* < 0.0001 for all), respectively]. Post-treatment nerve densities increased compared to pre-treatment to 5.3 (0.0–19.4, *p* = 0.0083) for total, 3.5 (0.0–13.2, *p* = 0.0059) for main, and 2.0 (0.0–10.4, *p* = 0.0251) for branch nerves, but remained lower than controls (*p* < 0.0001 for all). Corneal sensation increased from 2.3 ± 1.1 cm pre-treatment to 4.1 ± 1.4 cm post-treatment (*p* = 0.001). Median best corrected visual acuity significantly increased following rhNGF treatment from 0.4 (0.0–1.6) to 0.12 (−0.1 to 1.6) (*p* = 0.007).

**Conclusion:**

Patients with NK treated with at least 4 weeks of rhNGF, showed a significant increase in corneal nerve densities after treatment. A significant increase in corneal sensation, as well as best corrected visual acuity, was observed following treatment.

## 1. Introduction

Neurotrophic keratopathy (NK) is a rare neurodegenerative disease of the cornea with a prevalence of 5 in every 10,000 individuals that can lead to sight-threatening corneal ulcers and perforations if left untreated (Sacchetti and Lambiase, 2014; Dua et al., 2018). The cornea is one of the most densely innervated tissues in the body with sensory and autonomic nerve fibers, which are important in maintaining ocular surface health (Müller et al., 2003). Sensory corneal nerves are derived from the ophthalmic branch of the trigeminal nerve (Müller et al., 2003). After entering the cornea at the corneoscleral limbus, stromal nerve bundles create a dense subepithelial plexus (Belmonte et al., 2017). Nerves then penetrate the Bowman’s layer, the interface of the corneal stroma and epithelial layers, before branching and anastomosing to form the subbasal plexus, while superficial corneal nerves also contribute to this plexus (Marfurt et al., 2010). These nerves then terminate at the superficial layers of the epithelium (Marfurt et al., 2010). Corneal nerves are responsible for eliciting responses that maintain corneal homeostasis. Tear secretion from the lacrimal gland is a part of the trigeminal-parasympathetic reflex, a sensory reflex designed to protect the eye from harmful stimuli (Labetoulle et al., 2019). Corneal nerves also serve a trophic function with release of neuromediators and neurotrophins that maintain health of epithelial cells and keratocytes that in turn release growth factors and cytokines that initiate the regeneration of corneal nerves and epithelial cells (Al-Aqaba et al., 2019).

In patients with NK, the nerves and their function are impaired due to nerve damage and dysfunction (Bonini et al., 2003; Al-Aqaba et al., 2019). Nerve damage leads to changes in levels of neuromodulators and may result in reduced tear secretion, neurogenic inflammation, and impairment of epithelial cell health (Bonini et al., 2003; Dua et al., 2018; Al-Aqaba et al., 2019). The etiology of NK is most commonly due to herpetic keratitis, diabetes, dry eye disease, and surgical trauma (Versura et al., 2018; Bian et al., 2022; NaPier et al., 2022; Roszkowska et al., 2022). Depending on the severity of the ocular surface manifestations, NK is classified into three stages (Mackie et al., 1995; Sacchetti and Lambiase, 2014). Stage 1 is characterized by the presence of superficial punctate keratopathy, stage 2 is characterized by a persistent or reoccurring corneal epithelial defect, and stage 3 is characterized by the presence of a corneal ulcer (Mackie et al., 1995; Sacchetti and Lambiase, 2014).

Diagnosis of NK is traditionally made when patients show reduced corneal sensitivity (Bonini et al., 2003), quantified using the Cochet-Bonnet esthesiometer or a cotton wool whisp (Mandahl, 1993; Mantelli et al., 2015). More recently, *in vivo* confocal microscopy (IVCM) (HRT3/RCM, Heidelberg Engineering, Heidelberg, Germany) has allowed for the visualization of corneal nerves in several diseases, including NK, herpes simplex virus keratitis, herpes zoster keratitis, dry eye disease, and other types of infectious keratitis, among others (Hamrah et al., 2010, 2013; Cruzat et al., 2011, 2017; Alhatem et al., 2012; Sacchetti and Lambiase, 2014; Müller et al., 2015; Tepelus et al., 2017; Cavalcanti et al., 2018; Moein et al., 2018; Binotti et al., 2020). Multiple studies have previously shown reduced nerve fiber density in NK patients (Morishige et al., 1999; Xie et al., 2022; Yavuz Saricay et al., 2022). Thus, the imaging of the subbasal nerve plexus by IVCM allows for assessment of nerve density longitudinally (Cruzat et al., 2017).

Cenegermin 0.002% (Oxervate, Dompè Farmaceutici, Milan, Italy), a recombinant human nerve growth factor (rhNGF), is the first FDA approved drug for treatment of NK (Pflugfelder et al., 2020). The use of rhNGF for treatment of NK has been studied in two prospective, multicenter, randomized clinical trials for treatment of stage 2 and 3 NK (Bonini et al., 2018; Pflugfelder et al., 2020). These trials demonstrated efficacy in corneal healing post-treatment, however improvement in other clinical outcomes, such as corneal sensitivity and corneal nerve density have not been well demonstrated yet. A few studies have looked at nerve regeneration following rhNGF treatment and found a general increase, however these studies primarily report findings for stage 2 and 3 patients (*n* = 18) or few stage 1 patients (*n* = 5) (Mastropasqua et al., 2020; Yavuz Saricay et al., 2022). Corneal sensitivity improvements have also been identified following rhNGF treatment in stage 2 and 3 NK patients (Mastropasqua et al., 2020). Therefore, we aim to assess the effect of rhNGF treatment on corneal nerve regeneration, corneal sensitivity, and visual acuity in patients with NK stages 1, 2 and 3, with the largest group of NK stage 1 patients to date.

## 2. Materials and methods

### 2.1. Study design and patient selection

This is a retrospective, longitudinal, controlled single center study that was performed at the Cornea Service at the New England Eye Center, Tufts Medical Center, Boston, Massachusetts. Subjects were identified by conducting an electronic medical record search for patients seen at New England Eye Center, Boston, Massachusetts ranging from January 2015 to March 2022 for either of the following: (1) any patient diagnosed with NK via the ICD10 code for NK (H16.23X); or (2) those whose chart contained the keyword “neurotrophic.” To be included in the analysis, patients had to be above 18 years old, diagnosed with NK, and were required to have completed at least 4 weeks of rhNGF treatment for 6 times a day, with corresponding IVCM images before initiation of treatment and after treatment completion. The respective diagnosis criteria for each stage included 3+ central epithelial staining and decreased corneal sensation for stage 1, persistent epithelial defect for stage 2, and a corneal ulcer for stage 3 NK. Subjects were excluded if they had any evidence of active ocular infection or intraocular inflammation, any other ocular conditions that required topical medications, any history of severe systemic allergy or severe ocular allergy, any prior surgical treatment for NK (including tarsorrhaphy and conjunctival flap), use of contact lenses during the study period (refractive or therapeutic), use of botulinum toxin for blepharoptosis, or history of ocular surgery during rhNGF treatment or up to 3 months prior, lack of IVCM images for analysis, and concurrent start of autologous serum tears with rhNGF. If patients were treated with rhNGF in both eyes and fit the inclusion/exclusion criteria, one eye was randomly selected for analysis. Control IVCM images were selected from a database of healthy reference controls, which were collected for a previous prospectively enrolled study. These controls were required to have no symptoms, ocular surface disease, or ocular disease, not take any ocular medications, and have normal Schirmer’s test and tear break-up time. A subset of subjects from this database of healthy reference controls group were selected such that there was no significant difference between the groups in age or sex distribution.

Patient demographics, medications, surgical history, and prior medical history were collected from charts on the days of service. Documented clinical signs include tear break-up time, corneal fluorescein staining graded via the oxford scale (Bron et al., 2003), and tear production as assessed via Schirmer’s testing with anesthesia. Reported symptoms including blurry vision, sensitivity to light, irritation, foreign body sensation, burning, itchiness, pain, floaters, redness, discomfort, diplopia, dryness, tearing, and flashes were also documented when reported. Corneal sensitivity measurements were recorded as measured using the Cochet-Bonnet esthesiometer (Luneau Ophthalmologie, Chartres, France). Best corrected visual acuity was recorded (Snellen or ETDRS) and translated into LogMAR values (Holladay, 1997). Data was managed and organized through REDCap, with access provided through Tufts Medical Center (Harris et al., 2009, 2019).

### 2.2. *In vivo* confocal microscopy

IVCM was performed on the corneas of patients using Heidelberg Retina Tomograph 3 with the Rostock Cornea Module (Heidelberg Engineering GmbH, Heidelberg, Germany) as previously described (Cruzat et al., 2011). A drop of 0.5% proparacaine hydrochloride (Alcaine; Alcon, Fort Worth, TX) was instilled in the selected eye, followed by a drop of hydroxypropyl methylcellulose 2.5% (GenTeal gel, Alcon) in the selected eye. A disposable sterile polymethyl methacrylate cap (Tomo-Cap; Heidelberg Engineering GmbH) filled with drop of 2.5% hydroxypropyl methylcellulose (GenTeal gel) was mounted to the microscope. Another drop of 2.5% hydroxypropyl methylcellulose (GenTeal gel) was placed on the outside tip of Tomo-Cap to improve optical coupling. The microscope was manually moved until the Tomo-Cap was positioned on the corneal surface. After fixation of the patient’s gaze using a light for contralateral eye, the images were captured from the central apex. Images were taken focusing on the subbasal corneal nerves. This layer is at around 50 μm depth and anterior to the corneal stroma (Akhlaq et al., 2022). Images had been obtained for clinical evaluation before and after treatment.

### 2.3. Image analysis

Once eligible patients and selected eyes were identified, one masked observer (AB) selected the three most representative IVCM images that had good contrast and sharpness of focus for the study eye, which was randomly chosen if both eyes were affected, from both pre- and post-treatment visits. Two masked observers (AB and CSB) quantified the total, main, and branch corneal nerve densities and numbers in a semi-automated fashion using ImageJ software (Cruzat et al., 2011) (developed by Wayne Rasband, National Institutes of Health, Bethesda, MD; available at http://rsb.info.nih.gov.ezproxy.library.tufts.edu/ij/) with a semi-automated plug-in nerve tracing program NeuronJ.^1^ Main nerves were defined as a nerve that covers at least two thirds of an image and did not branch from another nerve. Branch nerves were defined as any nerves branching off of a main nerve or a nerve shorter than two thirds length of the image. Total nerves were defined as the sum of the main and branch nerves (Cruzat et al., 2011). Nerve densities and number were calculated by tracing visible nerves and were expressed in mm/mm^2^ and number/mm^2^, respectively. Total, main, and branch nerve densities and numbers were calculated for each image by averaging the two values from each observer. Average densities and numbers for each subject were then calculated from the values for all three images. Nerve regeneration rate per month was calculated using the following equation: (follow up total nerve density – baseline total nerve density)/weeks between visits for patients who had follow up within 7 days of ending treatment (multiplied by 4) (Mastropasqua et al., 2020).

### 2.4. Statistical analysis

For all data, normality was determined via Shapiro–Wilk test. Post-treatment corneal nerve densities and numbers were compared between controls and pre-treatment NK subjects using t-test/Mann–Whitney U tests as appropriate. Pre- and post-treatment differences were assessed via Wilcoxon signed ranked test, or paired t-test as appropriate. A receiver operating characteristic (ROC) curve analysis was used to assess nerve regeneration threshold for improvement in clinical signs and corneal sensitivity restoration. Correlations between clinical parameters and nerve parameters were assessed using Spearman’s rho test. All analyses were conducted using SPSS version 21.00 (SPSS, Inc., IBM, Armonk, NY) and GraphPad Prism version 9.4.1 (San Diego, CA).

## 3. Results

### 3.1. Patients and demographics

**Figure 1:**
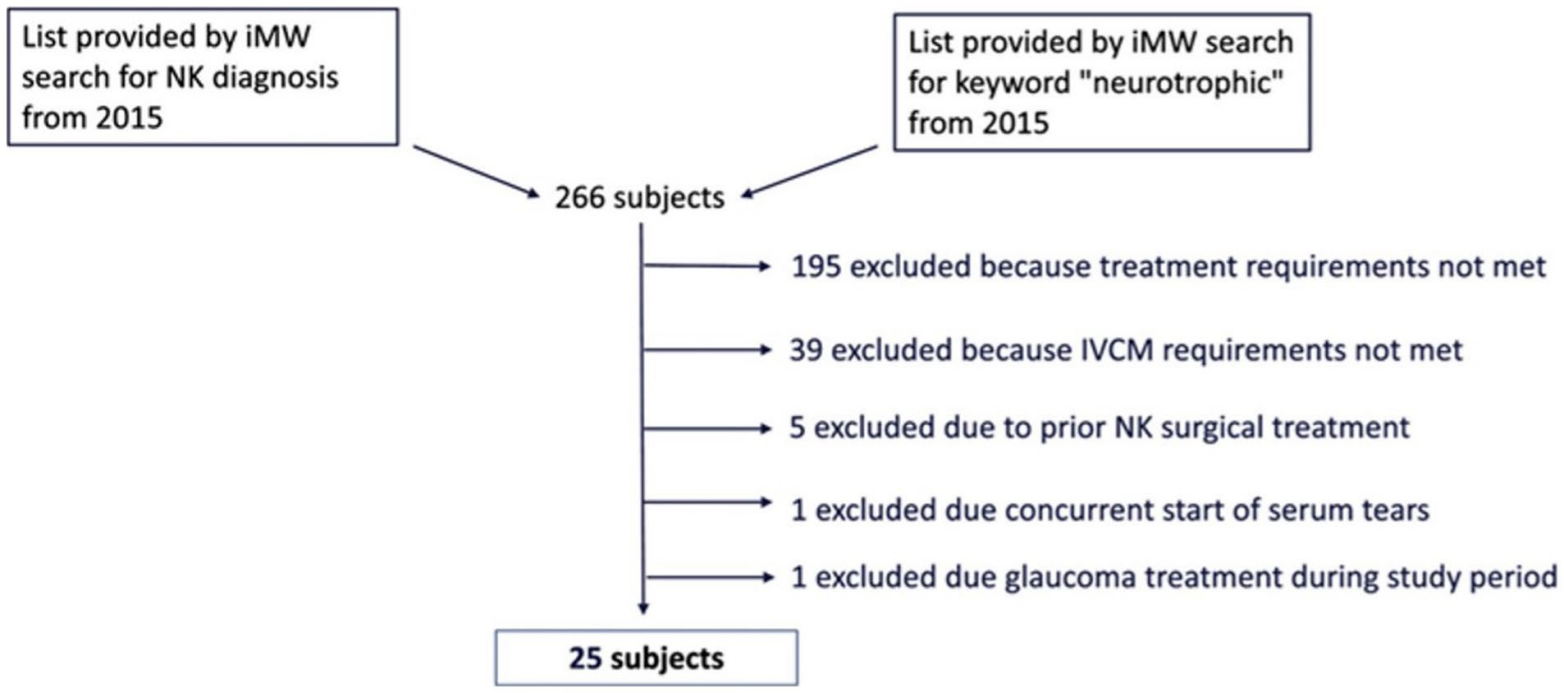
Breakdown of patient exclusion.

Our electronical medical record search identified 266 unique patients with NK. One hundred and ninety five of these subjects were excluded because they did not receive rhNGF treatment. Of 71 NK patients identified that were treated with rhNGF, 39 were excluded because they did not have the IVCM images available for analysis within the outlined timeframe. Five were excluded due to having prior surgical treatments for NK. One was excluded due to concurrent start of autologous serum tears, and another patient was excluded for receiving topical eye drops for glaucoma during the study period. Overall, this resulted in a sample of 25 NK subjects being included in our analysis (Figure 1); 88% of which had NK stage 1, 8% had NK stage 2, and 4% had NK stage 3. For demographics of patients please see Table 1. The median age of NK patients was 64 years (range: 30–93 years); subjects were 72% female, 96% white, and 92% were of non-Hispanic or Latino origin. However, 4% of subjects did not have a recorded ethnicity. The selected healthy reference control group included 20 subjects with a median age of 59 years (range: 49–74 years), and 40% were male. The NK and control groups did not differ in age (*p* = 0.10) or sex (*p* = 0.72). The most common prior medical history in NK patients was herpes simplex virus (HSV) keratitis and was seen in 48% of patients, followed by diabetes mellitus (36% of patients) (Table 2). The most common prior ocular surgery was cataract surgery (44% of patients). 96% of subjects completed 7–8 weeks of treatment, and 4% completed 4–6 weeks of treatment. Concurrent ocular treatments were recorded (Supplementary Table S1).

**Table 1:** Demographics of included patients with NK.

Parameter	Subjects
Age (years)	
Mean ± SD	64.2 ± 14.2
Median (range)	64 (30–93)
**Sex, *n* (%)**	
Female	18 (72%)
Male	7 (28%)
Race, *n* (%)	
White	24 (96%)
Asian	1 (4%)
Ethnic, *n* (%)	
Non-Hispanic or Latino	23(92%)
Hispanic or Latino	1(4%)
Unknown	1(4%)
NK stages, *n* (%)	
1	22 (88%)
2	2 (8%)
3	1 (4%)

**Table 2:** Baseline medical history summary.

Medical history at baseline	Subjects, *n* (%)
Herpes simplex virus (HSV)	12 (48.0%)
Diabetes mellitus (DM)	9 (36.0%)
Sjögren’s disease	3 (12.0%)
Thyroid disease	2 (8.0%)
Rheumatoid arthritis	1 (8.0%)
Trigeminal neuralgia	1 (4.0%)
Small fiber neuropathy (SFN)	1 (4.0%)
Systemic lupus erythematosus	1 (4.0%)
Migraine	1 (4.0%)
Hypertension	1 (4.0%)
Multiple sclerosis	1 (4.0%)
Graft vs. host disease	1 (4.0%)
Multiple myeloma/leukemia	1 (4.0%)
Parotid gland tumor	1 (4.0%)
Gastroparesis	1 (4.0%)
Interstitial cystitis	1 (4.0%)
Scleroderma	1 (4.0%)
Raynaud’s syndrome	1 (4.0%)
Lung transplant	1 (4.0%)

### 3.2. Corneal subbasal nerve alterations by *in vivo* confocal microscopy

**Figure 2:**
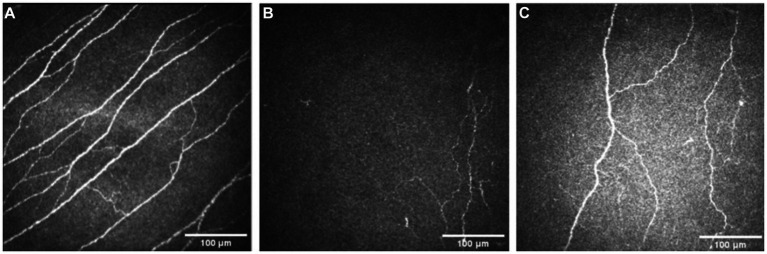
Examples of IVCM images from a healthy control **(A)**, and a patient with neurotrophic keratopathy pre- **(B)** and post-rhNGF treatment **(C)**.

**Figure 3:**
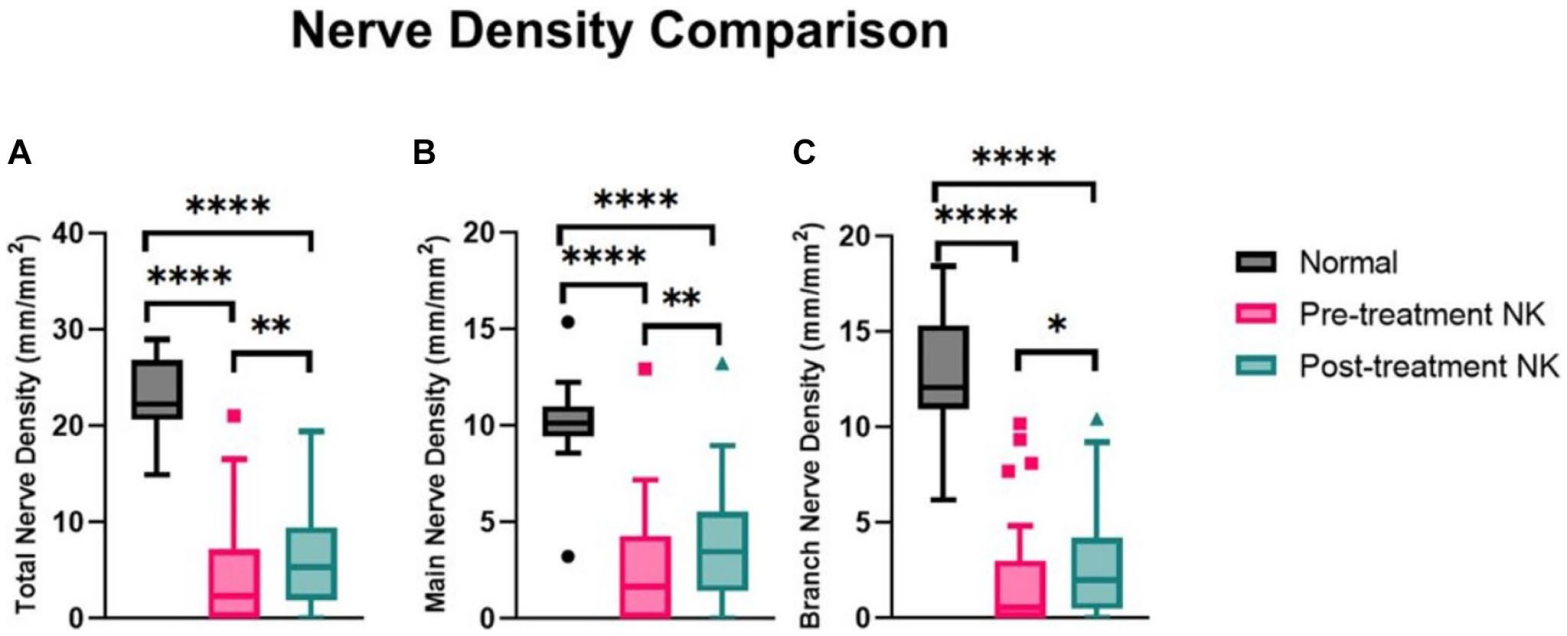
Median nerve density comparisons between pre- and post-rhNGF treatment and control groups for **(A)** total nerve density, **(B)** main nerve density, and **(C)** branch nerve density. Values presented in Tukey boxplot (****p < 0.0001, **p < 0.01, *p < 0.05).

**Figure 4:**
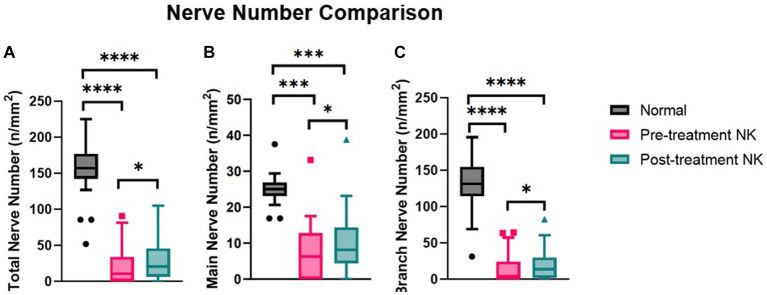
Median nerve number comparisons between pre- and post-rhNGF treatment and control groups for **(A)** total nerve number, **(B)** main nerve number, and **(C)** branch nerve number. Values presented in Tukey boxplot (***p < 0.001, **p < 0.01, *p < 0.05).

Pre-treatment median total nerve density was significantly lower in the NK group [2.3 mm/mm^2^ (range: 0.0–21.1)] compared to the total nerve density of the control group [22.3 mm/mm^2^ (range: 14.9–29.0), *p* < 0.0001] (Figures 2A–C, 3). Median main nerve density was also significantly lower in the NK group pre-treatment [1.7 mm/mm^2^ (range: 0.0–13.0)] compared to the main nerve density of the control group [10.1 mm/mm^2^ (range: 3.2–15.4), *p* < 0.0001]. Median branch nerve density was significantly lower in the NK group pre-treatment [0.5 mm/mm^2^ (range: 0.0–10.2)] compared to the control group [12.1 mm/mm^2^ (range: 6.2–18.4), *p* < 0.0001] (Figures 2A–C, 3, Table 3). Pre-treatment median total nerve number was significantly lower in the NK group [10.6 n/mm^2^ (range: 0.0–90.6)] compared to the total nerve number of the control group [157.5 n/mm^2^ (range: 51.9–225.0), *p* < 0.0001] (Figures 2A,B, 4). Median main nerve number was also significantly lower in the NK group pre-treatment [6.3 n/mm^2^ (range: 0.0–33.1)] compared to the main nerve number of the control group [25.0 n/mm^2^ (range: 16.9–37.5), *p* < 0.001]. Median branch nerve number was significantly lower in the NK group pre-treatment [4.4 n/mm^2^ (range: 0.0–64.4)] compared to the branch nerve number of the control group [131.3 n/mm^2^ (range: 31.3–195.6), *p* < 0.0001] (Figures 2A,B, 4, Table 3).

**Table 3:** Nerve density and number changes via IVCM measurements.

	Normal	Pre-treatment NK	Post-treatment NK	*p* value
Total nerve density in mm/mm^2^	22.7 ± 4.0; 22.3 (14.9–29.0)	4.6 ± 6.1; 2.3 (0.0–21.1)	6.4 ± 5.8; 5.3 (0.0–19.4)	^°^*p* < 0.0001 ^•^*p* < 0.0001 ^×^*p* = 0.0083
Main nerve density in mm/mm^2^	10.1 ± 2.2; 10.1 (3.2–15.4)	2.6 ± 3.2; 1.7 (0.0–13.0)	3.7 ± 3.3; 3.5 (0.0–13.2)	^°^*p* < 0.0001 ^•^*p* < 0.0001 ^×^*p* = 0.0059
Branch nerve density in mm/mm^2^	12.5 ± 3.2; 12.1 (6.2–18.4)	2.1 ± 3.3; 0.5 (0.0–10.2)	2.7 ± 2.8; 2.0 (0.0–10.4)	^°^*p* < 0.0001 ^•^*p* < 0.0001 ^×^*p* = 0.0251
Total nerve number in n/mm^2^	155.6 ± 44.4; 157.5 (51.9–225.0)	21.9 ± 28.8; 10.6 (0.0–90.6)	29.4 ± 28.8; 20.6 (0.0–105.0)	^°^*p* < 0.0001 ^•^*p* < 0.0001 ^×^*p* = 0.0118
Main nerve number in n/mm^2^	35.0 ± 46.3; 25.0 (16.9–37.5)	7.5 ± 8.1; 6.3 (0.0–33.1)	10.0 ± 8.8; 8.1 (0.0–38.8)	^°^*p* < 0.001 ^•^*p* < 0.001 ^×^*p* = 0.0162
Branch nerve number in n/mm^2^	130.6 ± 42.5; 131.3 (31.3–195.6)	14.4 ± 22.5; 4.4 (0.0–64.4)	19.4 ± 21.3; 13.8 (0.0–82.5)	^°^*p* < 0.0001 ^•^*p* < 0.0001 ^×^*p* = 0.0256

Results are presented as mean ± SD; median (range). Presented *p* values compare ^•^control vs pre-treatment NK, ^°^control vs post-treatment, ^×^pre-treatment vs post-treatment.

Compared to pre-treatment values in NK, median post-treatment total nerve density significantly increased to 5.3 mm/mm^2^ [(range: 0.0–19.4), *p* = 0.0083], main nerve density significantly increased to 3.5 mm/mm^2^ [(range: 0.0–13.2), *p* = 0.0059], and branch nerve density significantly increased to 2.0 mm/mm^2^ [(range: 0.0–10.4), *p* = 0.0251]. Median post-treatment total, main, and branch nerve densities were still significantly lower than controls (all *p* < 0.0001) (Figures 2A–C, 3, Table 3). The rate of nerve regeneration rate was calculated and was 0.8 ± 0.5 mm/mm^2^ per month. Compared to pre-treatment values in NK, median post-treatment total nerve number in n/mm^2^ increased significantly [20.6 (range: 0.0–105.0), *p* = 0.0118], main nerve number increased significantly [8.1 (range: 0.0–38.8), *p* = 0.0162], and branch nerve number increased significantly [13.8 (range: 0.0–82.5), *p* = 0.0256]. Median post-treatment total, main, and branch nerve numbers were still significantly lower than controls (all *p* < 0.0001) (Figures 2A–C, 4, Table 3).

Nerve density and number changes were stratified by severity stage of NK and analyzed (Table 4). Median total nerve density increased from 2.6 mm/mm^2^ (range: 0.0–21.1) to 6.0 mm/mm^2^ (range: 0.0–19.4) post-treatment in stage 1 NK (*p* = 0.021), from 2.2 mm/mm^2^ (range: 0.4–3.9) to 3.7 mm/mm^2^ (range: 2.1–5.3) post-treatment in stage 2 NK, and increased from 0.0 to 2.2 in one subject with stage 3 NK. Median main nerve density increased from 2.0 mm/mm^2^ (range: 0.0–12.9) to 3.7 mm/mm^2^ (range: 0.0–13.2) (*p* = 0.014) post-treatment in stage 1 NK, from 2.0 mm/mm^2^ (range: 0.4–3.6) to 2.3 mm/mm^2^ (range: 1.7–3.0) post-treatment in stage 2 NK, and increased from 0.0 to 1.7 mm/mm^2^ in one subject with stage 3 NK. Median branch nerve density increased from 0.7 mm/mm^2^ (range: 0.0–10.2) to 2.2 mm/mm^2^ (range: 0.0–10.4) (*p* = 0.070) post-treatment in stage 1 NK, from 0.2 mm/mm^2^ (range: 0.0–0.3) to 1.4 mm/mm^2^ (range: 0.5–2.3) post-treatment in stage 2 NK, and increased from 0.0 mm/mm^2^ to 0.5 mm/mm^2^ in one subject with stage 3 NK. Median total nerve number increased from 12.0 n/mm^2^ (range: 0.0–90.6) to 23.4 n/mm^2^ (range: 0.0–105.2) post-treatment in stage 1 NK (*p* = 0.028), from 6.3 n/mm^2^ (range: 2.1–10.4) to 14.0 n/mm^2^ (range: 6.3–21.9) post-treatment in stage 2 NK, and increased from 0.0 to 6.3 in one subject with stage 3 NK. Median main nerve number increased from 7.3 n/mm^2^ (range: 0.0–33.3) to 9.4 n/mm^2^ (range: 0.0–38.5) post-treatment in stage 1 NK (*p* = 0.041), from 5.2 n/mm^2^ (range: 2.1–8.3) to 6.3 n/mm^2^ (range: 4.2–8.3) post-treatment in stage 2 NK, and increased from 0.0 n/mm^2^ to 4.2 n/mm^2^ in one subject with stage 3 NK. Median branch nerve number increased from 5.7 n/mm^2^ (range: 0.0–64.6) to 15.1 n/mm^2^ (range: 0.0–82.3) post-treatment in stage 1 NK (*p* = 0.051), from 1.0 n/mm^2^ (range: 0.0–2.1) to 7.8 n/mm^2^ (range: 2.1–13.5) post-treatment in stage 2 NK, and increased from 0.0 n/mm^2^ to 2.1 n/mm^2^ in one subject with stage 3 NK.

**Table 4:** Stratification of nerve density and number by stages of neurotrophic keratopathy.

	Stage 1 NK (*n* = 22)			Stage 2 NK (*n* = 2)		Stage 3 NK (*n* = 1)	
	Pre-treatment	Post-treatment	*p* value	Pre-treatment	Post-treatment	Pre-treatment	Post-treatment
Total nerve density in mm/mm^2^	5.1 ± 6.4; 2.6 (0.0–21.1)	6.8 ± 6.1; 6.0 (0.0–19.4)	**0.021**	2.2 ± 2.5; 2.2 (0.4–3.9)	3.7 ± 2.3; 3.7 (2.1–5.3)	0.0	2.2
Main nerve density in mm/mm^2^	2.7 ± 3.3; 2.0 (0.0–12.9)	3.9 ± 3.4; 3.7 (0.0–13.2)	**0.014**	2.0 ± 2.2; 2.0 (0.4–3.6)	2.3 ± 1.0; 2.3 (1.7–3.0)	0.0	1.7
Branch nerve density in mm/mm^2^	2.4 ± 3.4; 0.7 (0.0–10.2)	2.9 ± 2.9; 2.2 (0.0–10.4)	0.070	0.2 ± 0.2; 0.2 (0.0–0.3)	1.4 ± 1.3; 1.4 (0.5–2.3)	0.0	0.5
Total nerve number in n/mm^2^	24.5 ± 30.0; 12.0 (0.0–90.6)	32.1 ± 29.6; 23.4 (0.0–105.2)	**0.028**	6.3 ± 5.9; 6.3 (2.1–10.4)	14.1 ± 11.0; 14.0 (6.3–21.9)	0.0	6.3
Main nerve number in n/mm^2^	8.0 ± 8.5; 7.3 (0.0–33.3)	10.7 ± 9.3; 9.4 (0.0–38.5)	**0.041**	5.2 ± 4.4; 5.2 (2.1–8.3)	6.3 ± 2.9; 6.3 (4.2–8.3)	0.0	4.2
Branch nerve number in n/mm^2^	16.4 ± 23.2; 5.7 (0.0–64.6)	21.0 ± 21.7; 15.1 (0.0–82.3)	0.051	1.0 ± 1.5; 1.0 (0.0–2.1)	7.8 ± 8.1; 7.8 (2.1–13.5)	0.0	2.1

Results are presented as mean ± SD; median (range).

### 3.3. Clinical parameters

**Figure 5:**
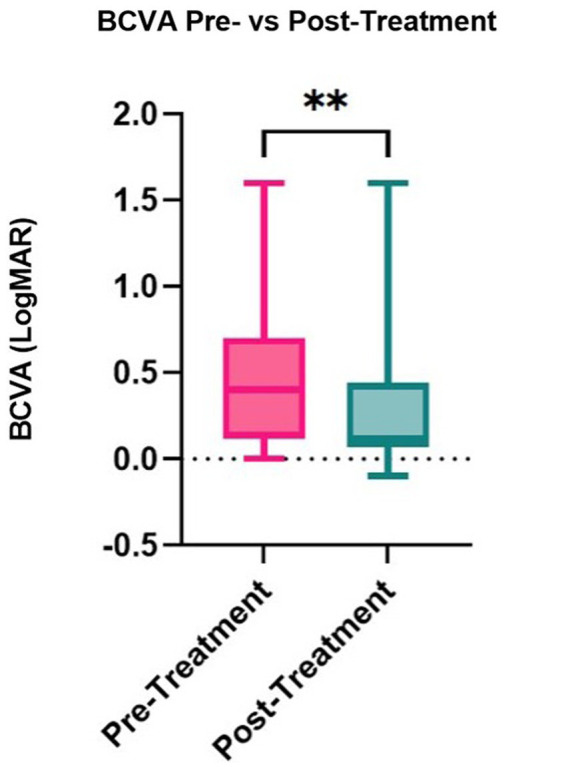
Box and whisker plot showing distribution BCVA pre- and post-rhNGF values (**p < 0.01).

Median corneal sensation improved significantly from 2.5 cm (range: 0.5–4.0) to 4.5 cm (range: 0.5–6.0) post-treatment (*p* = 0.001, Table 5), with an improvement in sensation seen in 92.86% of stage 1 NK patients. The median corneal fluorescein staining for stage 1 NK patients decreased significantly from 3.0 (range: 0.0–5.0) at baseline to 2.0 (range: 0.0–5.0) post-treatment (*p* = 0.001), with an improvement in fluorescein staining in 66.7% of stage 1 NK patients. Intraocular pressure, tear break-up time, and tear production measured by Schirmer’s test did not change significantly (*p* = 0.68, *p* = 0.49, and *p* = 0.058, respectively). Median best corrected visual acuity increased from LogMAR 0.4 (Snellen equivalent = 20/50; range: 0.0–1.6) to LogMAR 0.1 (Snellen equivalent = 20/25–1; range: −0.1 to 1.6; *p* = 0.007) (Figure 5).

**Table 5:** Summary of clinical findings pre- and post-rhNGF treatment.

Test	Pre-treatment	Post-treatment	*p* value
Corneal sensation (Cochet-Bonnet esthesiometry)	2.3 ± 1.1; 2.5 (0.5–4)	4.1 ± 1.4; 4.5 (0.5–6)	**0.001**
Corneal fluorescein staining (Oxford scale)	3.1 ± 1.4; 3.0 (0.0–5.0)	1.9 ± 1.3; 2.0 (0.0–5.0)	**0.001**
Tear break-up time (TBUT, seconds)	5.2 ± 2.4; 5.0 (2.0–10.0)	6.1 ± 4.1; 6.0 (0.0–18.0)	0.490
Intraocular pressure (IOP, mmHg)	16.1 ± 4.4; 16.0 (8.0–27.0)	15.8 ± 4.5; 15.0 (9.0–27.0)	0.680
Schirmer’s test with anesthesia (mm)	8.58 ± 6.0; 8.0 (1.0–16.0)	7.25 ± 6.6; 4.0 (1.0–16.0)	0.058
Best corrected visual acuity (logMAR)	0.4 ± 0.4; 0.4 (0–1.6)	0.3 ± 0.4; 0.1 (−0.1–1.6)	**0.007**

Results are presented as mean ± SD; median (range).

Bold values indicate significant *p* values.

Changes in self-reported ocular symptoms before and after treatment were also analyzed. Our results showed a reduction in the prevalence of blurry vision (44% prior to treatment to 20% post-treatment), foreign body sensation (24% pre-treatment to 16% post-treatment), burning (20% pre-treatment to 16% post-treatment), itchiness (16% pre-treatment to 12% post-treatment), floaters (12% pre-treatment to 4% post-treatment), and discomfort (8% pre-treatment to 4% post-treatment). Other symptoms completely resolved such as pain (12% pre-treatment), redness (12% pre-treatment), diplopia (8% pre-treatment), tearing (4% pre-treatment), and flashes (4% pre-treatment). We did not find any change in sensitivity to light, irritation, or dryness (Supplementary Table S2).

### 3.4. Nerve regeneration threshold

**Figure 6:**
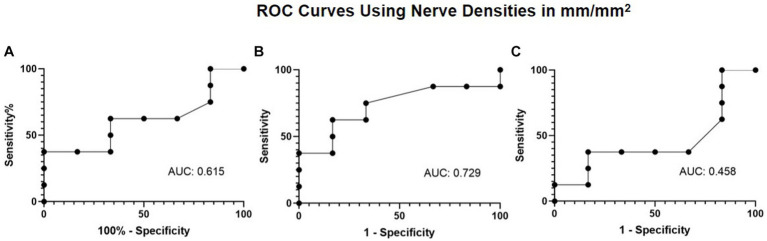
ROC curve using nerve densities in mm/mm^2^ for restoration of corneal sensation with **(A)** total nerve density, **(B)** main nerve density, and **(C)** branch nerve density.

Given the improvement observed in nerve density and corneal sensitivity, the association between nerve regeneration and corneal sensitivity was explored via a ROC curve. The ROC curve analysis aimed to evaluate the extent of total, main, and branch nerve regeneration necessary to increase corneal sensitivity by 1.5 cm or more. The resulting area under the curve (AUC) values were 0.615, 0.729, and 0.458 for total, main, and branch, respectively (Figure 6). A nerve regeneration threshold was determined via the main nerve density change, because it provided the highest AUC value. A cut-off value of 0.622 mm/mm^2^ provides a 0.625 sensitivity and a 0.833 specificity. This suggests that of patients who experience 1.5 cm or more increase in corneal sensitivity, approximately 62.5% will have had an increase in main nerve density of 0.622 mm/mm^2^ or more increase and that of patients who did not experience 1.5 cm or more increase in corneal sensitivity, approximately 83.3% will have less than 0.622 mm/mm^2^ main nerve density increase.

**Figure 7:**
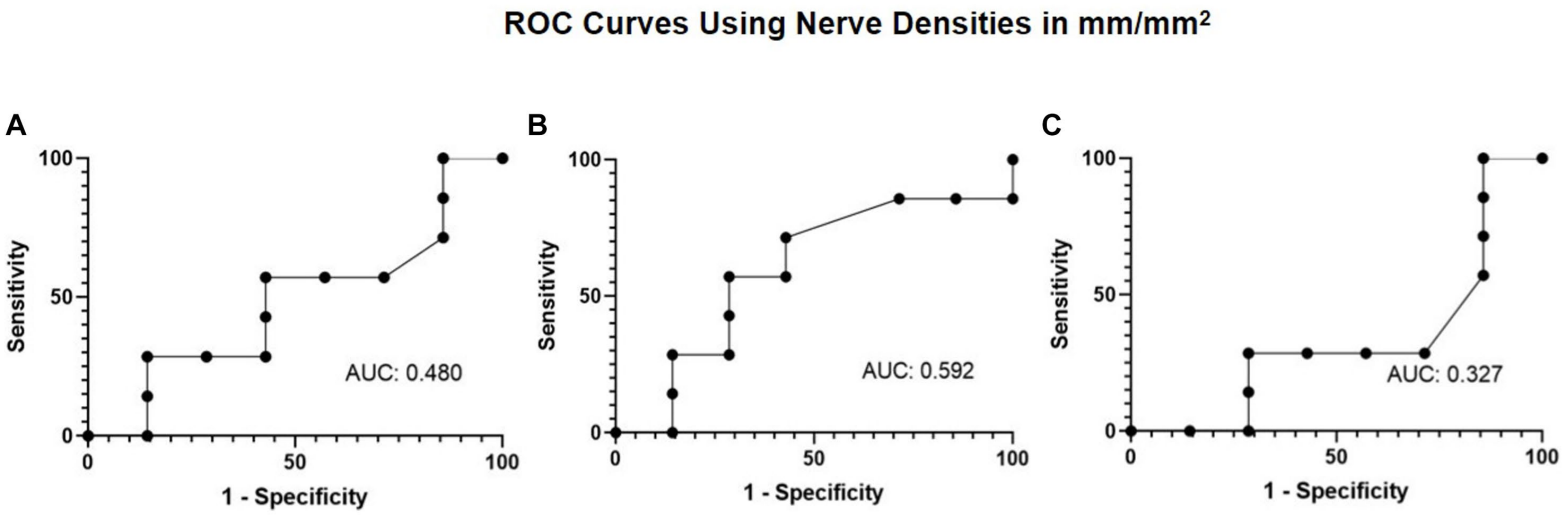
ROC curve using nerve densities in mm/mm^2^ for improvement in corneal fluorescein staining with **(A)** total nerve density, **(B)** main nerve density, and **(C)** branch nerve density.

Given there was also a decrease in corneal fluorescein staining with treatment, ROC curves were generated to explore the association of nerve density and corneal fluorescein staining. The ROC curve analysis aimed to evaluate the extent of total, main, and branch nerve regeneration necessary to decrease corneal staining by a grade of 1 or more. The resulting area under the curve (AUC) values of 0.480, 0.592, and 0.327 for total, main, and branch, respectively (Figure 7). A nerve regeneration threshold was determined via the main nerve density change because it provided the highest AUC value. This cut-off value of 0.622 mm/mm^2^ suggests that an increase of 0.622 mm/mm^2^ in main nerve density will provide a decrease in corneal staining of one with a 0.571 sensitivity, and a 0.714 specificity. This suggests that of patients who experience an improvement of 1 in corneal staining or more, approximately 57.1% will have had an increase in main nerve density of 0.622 mm/mm^2^ or more increase and that of patients who did not experience an improvement of 1or more increase in corneal staining, approximately 71.4% will have less than 0.622 mm/mm^2^ main nerve density increase.

### 3.5. Correlation analysis

**Figure 8:**
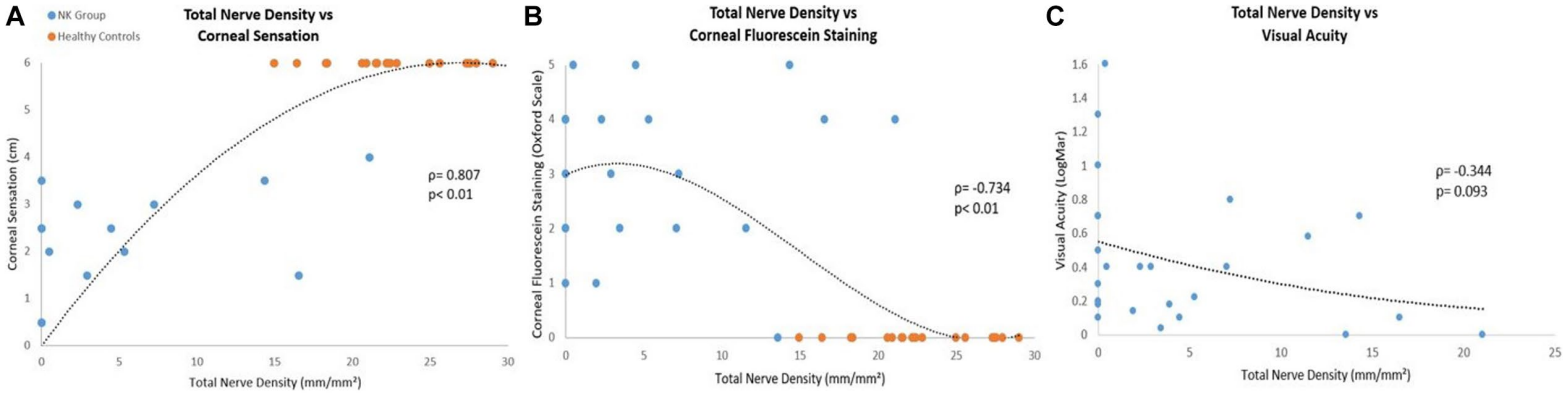
Correlations between pre-rhNGF treatment and control groups total nerve density with **(A)** corneal sensation, **(B)** corneal fluorescein staining, and **(C)** visual acuity. Visual acuity measurements were not available for control groups. Both *ρ* and *p* values are presented.

We analyzed correlations between clinical parameters and nerve parameters (Table 6, Figure 8). There were significant positive correlations between corneal sensation and nerve parameters [total nerve density (*ρ* = 0.807) and number (*ρ* = 0.822); main nerve density (*ρ* =0.746) and number (*ρ* = 0.765); branch nerve density (*ρ* = 0.810) and number (*ρ* = 0.829)] (all *p* < 0.01). There were also significant correlations between changes in corneal fluorescein staining and nerve parameters [total nerve density (*ρ* = −0.734) and number (*ρ* = −0.739); main nerve density (*ρ* = −0.660) and number (*ρ* = −0.661) and changes in branch nerve density (*ρ* = −0.745), and number (*ρ* = −0.756)] (all *p* < 0.01). There were negative correlations between changes in best corrected visual acuity and nerve parameters [total nerve density (*ρ* = −0.344, *p* = 0.0093) and number (*ρ* = −0.375, *p* = 0.065); main nerve density (*ρ* = −0.337, *p* = 0.099) and number (*ρ* = −0.372, *p* = 0.067); branch nerve density (*ρ* = −0.347, *p* = 0.089) and number (*ρ* = −0.366, *p* = 0.072)], although these were not significant.

**Table 6:** Correlations between clinical findings and nerve parameters in patients with NK pre-rhNGF treatment and healthy controls.

	Corneal sensation		Corneal fluorescein staining		logMAR Visual acuity	
	*ρ* value	*p* value	*ρ* value	*p* value	*ρ* value	*p* value
Total nerve density in mm/mm^2^	0.807	**<0.01**	−0.734	**<0.01**	−0.344	0.093
Main nerve density in mm/mm^2^	0.746	**<0.01**	−0.660	**<0.01**	−0.337	0.099
Branch nerve density in mm/mm^2^	0810	**<0.01**	−0.745	**<0.01**	−0.347	0.089
Total nerve number in n/mm^2^	0.822	**<0.01**	−0.739	**<0.01**	−0.372	0.065
Main nerve number in n/mm^2^	0.765	**<0.01**	−0.661	**<0.01**	−0.372	0.067
Branch nerve number in n/mm^2^	0.829	**<0.01**	−0.756	**<0.01**	−0.366	0.072

Bold values indicate significant *p* values.

## 4. Discussion

This study found that rhNGF treatment in NK patients resulted in increased corneal nerve density and increased corneal sensitivity. Further, treatment resulted in improved vision and corneal fluorescein staining in stage 1 NK patients. Our sample consisted of 25 patients that were mostly white and of non-Hispanic or Latino origin, which is consistent with the demographic distribution reported previously for NK by the Intelligent Research in Sight (IRIS) registry (Bian et al., 2022). Our results also showed that HSV keratitis was the most common underlying condition in our sample of NK subjects (48%), independent of the disease staging, followed by diabetes mellitus (36%). This etiological finding is consistent with the most common etiologies identified in prior literature (Versura et al., 2018; Bian et al., 2022; NaPier et al., 2022; Roszkowska et al., 2022). These consistencies in demographics and etiology suggest that our results are likely generalizable to the broader NK population.

The significant improvements in total, main, and branch nerve densities following rhNGF treatment with accompanying improvements in sensation, corneal fluorescein staining, and vision underscore the importance of corneal nerves to ocular surface health and homeostasis. Improvements in nerve densities with rhNGF treatment are consistent with reports observed in a case series describing IVCM findings with rhNGF treatment in 5 out of 17 stage 1 NK patients (Yavuz Saricay et al., 2022) and in a study of 18 patients with stage 2 and 3 NK treated with rhNGF (Mastropasqua et al., 2020). In addition, our calculated rate of nerve regeneration, 0.84 ± 0.51 mm/mm^2^ per month after 8 weeks of treatment was within the range of those previously reported (between 0.6619 and 1.8700 mm/mm^2^ per month) (Mastropasqua et al., 2020; Yavuz Saricay et al., 2022). The two largest clinical trials with rhNGF, the NGF0214 study (*n* = 48) and the European REPARO study (*n* = 156), evaluated the efficacy of rhNGF in treatment of stage 2 and 3 NK (Bonini et al., 2018; Pflugfelder et al., 2020) and did not include any corneal nerve density analysis, but did show improvements in corneal epithelial surface integrity. Specifically, the NGF0214 study demonstrated that a greater percentage of subjects within the group treated with rhNGF showed resolution of their corneal defect compared to the placebo group (Pflugfelder et al., 2020), and the REPARO clinical trial found significance in corneal healing following both four and eight weeks of rhNGF treatment compared to vehicle treatment (Bonini et al., 2018). These improvements in corneal epithelial surface integrity are consistent with the improvements seen within our study via corneal staining. Recombinant human nerve growth factor has been established as a relevant treatment of corneal surface defects, as nerve growth factor is a neurotrophin that has been established to be present in the cornea, along with neurotrophins NT-3, NT-4 and BDNF, all encoded by the neurotrophin gene (You et al., 2000). Recently, Pedrotti et al. investigated corneal nerve alterations and changes in corneal sensitivity following cenegermin treatment in 18 patients with stage 2 and 3 NK, but not stage 1 NK. Their findings showed a significant increase in corneal nerve branch density starting at 2 months and corneal nerve fiber length starting at 8 weeks, in the central and cornea during the initial 4 months follow-up. However, there was no increase in corneal branch nerve densities within the central cornea after the 8 week treatment or the 8 month follow-up (Pedrotti et al., 2022). Our study explores these parameters as well as total and main nerve density in the central cornea in the largest sample size of stage 1 NK to date, and finds significance. We establish an increase in corneal sensation in those with stage 1 NK, and an increase in corneal nerve densities, expanding upon the prior findings of nerve regeneration and restoration of corneal sensation in stage 2 and 3 NK in the prior literature.

Our data shows that pre-treatment corneal nerve numbers and density were significantly reduced in patients with NK compared to healthy controls. Additionally, as NK progressed, the corneal nerves densities were more compromised, resulting in greater loss of corneal nerves on IVCM images at baseline as seen in nerve density values by stratification of stage. Moreover, branch nerve densities of patients at stage 1 and 2 NK showed a greater loss than main nerve density values compared to controls, IVCM images of the patient at stage 3 showed a complete loss of nerves. However, despite significant nerve regeneration in patients with all three stages, corneal nerve densities did not fully recover and remained significantly lower compared to healthy controls. TrkA receptors have been identified as NGF receptors located amongst the ocular surface and cornea (You et al., 2000; Gupta et al., 2022). NGF is hypothesized to induce nerve regeneration through activation of Schwann cells, induction of myelin, and axonal regeneration (Li et al., 2020). NGF has been shown to induce nerve regeneration via TrkA (de Castro et al., 1998). Further, animal models of TrkA knockout receptors have been found to have impairment of corneal nerves with corneal neuropathies (Smeyne et al., 1994; de Castro et al., 1998). Thus, the relatively limited improvement in corneal nerve densities without treatment with rhNGF treatment may potentially be attributed to the reduced expression of TrkA receptors due to severe loss of nerves. Furthermore, main nerves showed a greater increase in density than branch nerves post-treatment, suggesting main nerve regeneration contributed more than branch regeneration in the observed increase in total nerve density.

Interestingly, our study reported an improvement in corneal sensitivity with rhNGF treatment. Corneal sensitivity improvement after rhNGF treatment did not reach statistical significance in the REPARO and NGF0214 trials. However, other studies have shown significant increases in corneal sensitivity in moderate and severe NK cases after 8 weeks of rhNGF (Mastropasqua et al., 2020; Pedrotti et al., 2022; Roszkowska et al., 2022). It should be noted that our study included a large proportion of stage 1 NK patients compared to the other studies that exclusively assessed corneal sensitivity in stage 2 and 3 NK patients. We also found a significant improvement in BCVA following treatment, supporting the previous finding that BCVA improves following rhNGF treatment in stage 1 NK (Yavuz Saricay et al., 2022). In contrast to these findings, the NGF0214 study and REPARO study did not find any significant improvement in BCVA, but this difference is likely due to the representation of stages within the studies. Therefore, we conclude that rhNGF treatment in earlier stages of NK may increase the potential for improvement in BCVA.

Topical rhNGF is usually well-tolerated and its safety profile has been demonstrated in clinical trials (Bonini et al., 2018; Pflugfelder et al., 2020). A qualitative analysis of symptoms prevalence in our study showed a complete resolution of pain, redness, diplopia, tearing and flashes and a reduction of reported blurry vision, foreign body sensation, burning, itchiness, floaters, and discomfort. Researchers of the REPARO trial showed that the most relevant side effect of topical rhNGF is transient ocular pain, where ocular pain was reported in 7.7% of the NK study group and 3.8% of the vehicle control group (Bonini et al., 2018). Interestingly, our results showed a trend of pain reduction as 12% of our subjects experienced pain before treatment, which resolved completely post-treatment. Previous studies have shown that patients with evoked and spontaneous pain demonstrate a decrease in nerve density (Devigili et al., 2008; Aggarwal et al., 2019). Pain can result from inflammatory responses triggered by nerve damage (neurogenic inflammation), further resulting in a change of ion channel transduction, which upregulate ion channels in nociceptors, modify intrinsic membrane potentials, and activate nerve generating ectopic impulses (Aggarwal et al., 2019). Polymodal pain receptors are found to fire at a lower threshold due to the inflammatory responses following tissue injury (Goyal and Hamrah, 2016). This creates an increased sensitivity to noxious stimuli, called sensitization, which can result in allodynia and hyperalgesia (Kerstman et al., 2013). In addition, neurodegeneration processes are triggered by the interaction between neuronal and glial cells (Lobsiger and Cleveland, 2007). NGF has been identified as a modulator of glial reactions, which are hallmarks of pathological pain (Colangelo et al., 2008). Animal studies have demonstrated that NGF decreases allodynia and hyperalgesia by decreasing glial reactions elicited by peripheral nerve damage, and reversing neuromodulation at molecular and morphological level of peripheral sensory neurons (Colangelo et al., 2008; Cirillo et al., 2010). Reduction of pain in patients with NK treated with rhNGF may be due to the effect NGF has on nerves, as NGF has been demonstrated to play a role in the differentiation, proliferation and survival of sensory and sympathetic neurons in animal models (Colangelo et al., 2008; Cirillo et al., 2010). Similar to rhNGF, autologous serum tears contain neurotrophic and epithelial growth factors, such as NGF, insulin-like growth factor-1, transforming growth factor β, fibronectin, and epidermal growth factors (Di Fausto et al., 2007; Pan et al., 2017), which have a role on nerve regeneration through restoration of damaged neurons and induction of neuronal sprouting (Kerstman et al., 2013). Moreover, Aggarwal et al. (2019) demonstrated a significant reduction of pain after treatment of autologous serum tears in patients with neuropathic corneal pain. Thus, rhNGF treatment might improve pain scores through nerve regeneration and growth of sensory neurons, and consequently may improve the quality of life of NK patients.

Our ROC analysis suggests that improvement in main nerve density is needed to restore corneal sensitivity by a value of 1.5 cm and decrease corneal fluorescein staining by a grade of 1. Despite our results showing an acceptable cutoff for specificity but not for sensitivity, it suggests a strong relationship between a baseline requirement of main nerve regeneration for restoration of sensation as well as an improvement in staining. We hypothesize that nerve regeneration allows for improved corneal sensation due to restoration of corneal nerve function following increased density of nerves. We also hypothesize that as corneal nerves regenerate and increase in density, there is associated improvement in the health of the ocular surface, resulting in decreased staining.

Our correlational analysis shows correlations between the clinical parameters (corneal sensation, visual acuity, and corneal fluorescein staining), and nerve parameters (total, main, and branch densities and total and branch nerve numbers). Analysis shows a strong positive correlation for total, main, and branch nerve density and number, with corneal sensation and corneal fluorescein staining. This suggests that that as nerve density and number increases, there is improvement in corneal sensation and less corneal staining. Analysis also shows a negative correlation between visual acuity in LogMAR units and all nerve parameters. Although this was not significant, we hypothesize there may be a relationship between visual acuity and nerve parameters due to correlational findings. A decrease in logMAR visual acuity represents an improvement in visual acuity, suggesting that as nerve density and number increase, visual acuity improves. These findings suggest there may be a relationship between nerve parameters and clinical parameters, where higher nerve density and number correlate to lower corneal staining and better corneal sensitivity. We hypothesize this may be also due to improvement of nerve function with a greater nerve density and count.

Despite demonstrating significant improvement in nerve regeneration and other clinical outcomes, our study has several limitations. The retrospective nature of this study limited the acquisition of full data related to clinical signs for all subjects. Moreover, our sample size did not permit the assessment of treatment effects to be controlled by age, sex, race, or ethnicity, and did not allow for statistical comparison of clinical outcomes between groups of different stages. Another limitation is that most of our subjects had stage 1 NK, thus the novel finding of improved corneal sensation in stage 1 NK may not be applicable moderate and severe cases. Nevertheless, our report is the largest to date with 22 stage 1 NK patients, presenting data on corneal nerve density and sensitivity changes with rhNGF treatment in stage 1 NK. This allows us to expand upon previous clinical findings following rhNGF treatment for those with stage 2 and 3 NK (Mastropasqua et al., 2020). Moreover, various ocular surface disorders, such as dry eye disease, neuropathic corneal pain, limbal stem cell deficiency, and post-surgical conditions (such as laser- assisted *in situ* keratomileusies, photorefractive keratectomy, and penetrating keratoplasty) have demonstrated decreased corneal nerve densities (Calvillo et al., 2004; Erie et al., 2005; Beni’tez-del-Castillo et al., 2007; Patel et al., 2007; Deng et al., 2012; Aggarwal et al., 2015). Given that our study demonstrated efficacy of rhNGF in promoting corneal nerve regeneration, other conditions that have shown decreased corneal nerves may potentially benefit from rhNGF treatment.

In conclusion, this study shows that rhNGF was effective in regenerating corneal nerves and improving corneal sensation, a novel finding in patients with neurotrophic keratopathy. Improvement in corneal sensation following rhNGF treatment is a finding that is encouraging for clinical treatment.

## Data availability statement

The raw data supporting the conclusions of this article will be made available by the authors, without undue reservation.

## Ethics statement

The studies involving humans were approved by Tufts Institutional Review Board. The studies were conducted in accordance with the local legislation and institutional requirements. Written informed consent for participation was not required from the participants or the participants’ legal guardians/next of kin because this was a retrospective study.

## Author contributions

PH: conception and design, obtained funding, and overall responsibility. AB-P, CSB, and SMC: analysis and interpretation. AB-P and CSB: data collection. All authors contributed to the article and approved the submitted version.

## Footnotes

^1^ http://www.imagescience.org/meijering/software/neuronj/

## References

[ref1] Aggarwal S. Colon C. Kheirkhah A. Hamrah P. (2019). Efficacy of autologous serum tears for treatment of neuropathic corneal pain. Ocul. Surf. 17, 532–539. doi: 10.1016/j.jtos.2019.01.009, PMID: 30685437 PMC6956846

[ref2] Aggarwal S. Kheirkhah A. Cavalcanti B. M. Cruzat A. Colon C. Brown E.. (2015). Autologous serum tears for treatment of Photoallodynia in patients with corneal neuropathy: efficacy and evaluation with in vivo confocal microscopy. Ocul. Surf. 13, 250–262. doi: 10.1016/j.jtos.2015.01.005, PMID: 26045233 PMC4499014

[ref3] Akhlaq A. Colón C. Cavalcanti B. M. Aggarwal S. Qazi Y. Cruzat A.. (2022). Density and distribution of dendritiform cells in the peripheral cornea of healthy subjects using in vivo confocal microscopy. Ocul. Surf. 26, 157–165. doi: 10.1016/j.jtos.2022.07.008, PMID: 35998820

[ref4] al-Aqaba M. A. Dhillon V. K. Mohammed I. Said D. G. Dua H. S. (2019). Corneal nerves in health and disease. Prog. Retin. Eye Res. 73:100762. doi: 10.1016/j.preteyeres.2019.05.003, PMID: 31075321

[ref5] Alhatem A. Cavalcanti B. Hamrah P. (2012). In vivo confocal microscopy in dry eye disease and related conditions. Semin. Ophthalmol. 27, 138–148. doi: 10.3109/08820538.2012.711416, PMID: 23163268 PMC4456189

[ref6] Belmonte C. Nichols J. J. Cox S. M. Brock J. A. Begley C. G. Bereiter D. A.. (2017). TFOS DEWS II pain and sensation report. Ocul. Surf. 15, 404–437. doi: 10.1016/j.jtos.2017.05.002, PMID: 28736339 PMC5706540

[ref7] Beni’tez-del-Castillo J. M. Acosta M. C. Wassfi M. A. di’az-Valle D. Gegu’ndez J. A. Fernandez C.. (2007). Relation between corneal innervation with confocal microscopy and corneal sensitivity with noncontact esthesiometry in patients with dry eye. Invest. Ophthalmol. Vis. Sci. 48, 173–181. doi: 10.1167/iovs.06-0127, PMID: 17197530

[ref8] Bian Y. Ma K. K. Hall N. E. Elze T. Lorch A. Miller J. W.. (2022). Neurotrophic keratopathy in the United States: an intelligent research in sight registry analysis. Ophthalmology 129, 1255–1262. doi: 10.1016/j.ophtha.2022.06.019, PMID: 35768054

[ref9] Binotti W. W. Bayraktutar B. Ozmen M. C. Cox S. M. Hamrah P. (2020). A review of imaging biomarkers of the ocular surface. Eye Contact Lens 46, S84–s105. doi: 10.1097/ICL.0000000000000684, PMID: 31833999 PMC7354708

[ref10] Bonini S. Lambiase A. Rama P. Sinigaglia F. Allegretti M. Chao W.. (2018). Phase II randomized, double-masked, vehicle-controlled trial of recombinant human nerve growth factor for neurotrophic keratitis. Ophthalmology 125, 1332–1343. doi: 10.1016/j.ophtha.2018.02.022, PMID: 29653858

[ref11] Bonini S. Rama P. Olzi D. Lambiase A. (2003). Neurotrophic keratitis. Eye (Lond.) 17, 989–995. doi: 10.1038/sj.eye.6700616, PMID: 14631406

[ref12] Bron A. J. Evans V. E. Smith J. A. (2003). Grading of corneal and conjunctival staining in the context of other dry eye tests. Cornea 22, 640–650. doi: 10.1097/00003226-200310000-00008, PMID: 14508260

[ref13] Calvillo M. P. McLaren J. W. Hodge D. O. Bourne W. M. (2004). Corneal reinnervation after LASIK: prospective 3-year longitudinal study. Invest. Ophthalmol. Vis. Sci. 45, 3991–3996. doi: 10.1167/iovs.04-0561, PMID: 15505047

[ref14] Cavalcanti B. M. Cruzat A. Sahin A. Pavan-Langston D. Samayoa E. Hamrah P. (2018). In vivo confocal microscopy detects bilateral changes of corneal immune cells and nerves in unilateral herpes zoster ophthalmicus. Ocul. Surf. 16, 101–111. doi: 10.1016/j.jtos.2017.09.004, PMID: 28923503 PMC5756670

[ref15] Cirillo G. Cavaliere C. Bianco M. R. de Simone A. Colangelo A. M. Sellitti S.. (2010). Intrathecal NGF administration reduces reactive astrocytosis and changes neurotrophin receptors expression pattern in a rat model of neuropathic pain. Cell. Mol. Neurobiol. 30, 51–62. doi: 10.1007/s10571-009-9430-2, PMID: 19585233 PMC11498408

[ref16] Colangelo A. M. Bianco M. R. Vitagliano L. Cavaliere C. Cirillo G. de Gioia L.. (2008). A new nerve growth factor-mimetic peptide active on neuropathic pain in rats. J. Neurosci. 28, 2698–2709. doi: 10.1523/JNEUROSCI.5201-07.2008, PMID: 18337399 PMC6670672

[ref17] Cruzat A. Qazi Y. Hamrah P. (2017). In vivo confocal microscopy of corneal nerves in health and disease. Ocul. Surf. 15, 15–47. doi: 10.1016/j.jtos.2016.09.004, PMID: 27771327 PMC5512932

[ref18] Cruzat A. Witkin D. Baniasadi N. Zheng L. Ciolino J. B. Jurkunas U. V.. (2011). Inflammation and the nervous system: the connection in the cornea in patients with infectious keratitis. Invest. Ophthalmol. Vis. Sci. 52, 5136–5143. doi: 10.1167/iovs.10-7048, PMID: 21460259 PMC3176064

[ref19] de Castro F. Silos-Santiago I. de Armentia M. L. Barbacid M. Belmonte C. (1998). Corneal innervation and sensitivity to noxious stimuli in trkA knockout mice. Eur. J. Neurosci. 10, 146–152. doi: 10.1046/j.1460-9568.1998.00037.x, PMID: 9753121

[ref20] Deng S. X. Sejpal K. D. Tang Q. Aldave A. J. Lee O. L. Yu F. (2012). Characterization of limbal stem cell deficiency by in vivo laser scanning confocal microscopy: a microstructural approach. Arch. Ophthalmol. 130, 440–445. doi: 10.1001/archophthalmol.2011.378, PMID: 22159172 PMC3928362

[ref21] Devigili G. Tugnoli V. Penza P. Camozzi F. Lombardi R. Melli G.. (2008). The diagnostic criteria for small fibre neuropathy: from symptoms to neuropathology. Brain 131, 1912–1925. doi: 10.1093/brain/awn093, PMID: 18524793 PMC2442424

[ref22] di Fausto V. Fiore M. Tirassa P. Lambiase A. Aloe L. (2007). Eye drop NGF administration promotes the recovery of chemically injured cholinergic neurons of adult mouse forebrain. Eur. J. Neurosci. 26, 2473–2480. doi: 10.1111/j.1460-9568.2007.05883.x, PMID: 17970722

[ref23] Dua H. S. Said D. G. Messmer E. M. Rolando M. Benitez-del-Castillo J. M. Hossain P. N.. (2018). Neurotrophic keratopathy. Prog. Retin. Eye Res. 66, 107–131. doi: 10.1016/j.preteyeres.2018.04.003, PMID: 29698813

[ref24] Erie J. C. McLaren J. W. Hodge D. O. Bourne W. M. (2005). Recovery of corneal subbasal nerve density after PRK and LASIK. Am J. Ophthalmol. 140, 1059–1064.e1. doi: 10.1016/j.ajo.2005.07.027, PMID: 16376651

[ref25] Goyal S. Hamrah P. (2016). Understanding neuropathic corneal pain--gaps and current therapeutic approaches. Semin. Ophthalmol. 31, 59–70. doi: 10.3109/08820538.2015.1114853, PMID: 26959131 PMC5607443

[ref26] Gupta A. Galletti J. G. Yu Z. Burgess K. de Paiva C. S. (2022). A, B, C’s of Trk receptors and their ligands in ocular repair. Int. J. Mol. Sci. 23:14069. doi: 10.3390/ijms232214069, PMID: 36430547 PMC9695972

[ref27] Hamrah P. Cruzat A. Dastjerdi M. H. Prüss H. Zheng L. Shahatit B. M.. (2013). Unilateral herpes zoster ophthalmicus results in bilateral corneal nerve alteration: an in vivo confocal microscopy study. Ophthalmology 120, 40–47. doi: 10.1016/j.ophtha.2012.07.036, PMID: 22999636 PMC3575640

[ref28] Hamrah P. Cruzat A. Dastjerdi M. H. Zheng L. Shahatit B. M. Bayhan H. A.. (2010). Corneal sensation and subbasal nerve alterations in patients with herpes simplex keratitis: an in vivo confocal microscopy study. Ophthalmology 117, 1930–1936. doi: 10.1016/j.ophtha.2010.07.010, PMID: 20810171 PMC2949523

[ref29] Harris P. A. Taylor R. Minor B. L. Elliott V. Fernandez M. O’Neal L.. (2019). The REDCap consortium: building an international community of software platform partners. J. Biomed. Inform. 95:103208. doi: 10.1016/j.jbi.2019.103208, PMID: 31078660 PMC7254481

[ref30] Harris P. A. Taylor R. Thielke R. Payne J. Gonzalez N. Conde J. G. (2009). Research electronic data capture (REDCap)--a metadata-driven methodology and workflow process for providing translational research informatics support. J. Biomed. Inform. 42, 377–381. doi: 10.1016/j.jbi.2008.08.010, PMID: 18929686 PMC2700030

[ref31] Holladay J. T. (1997). Proper method for calculating average visual acuity. J. Refract. Surg. 13, 388–391. doi: 10.3928/1081-597X-19970701-16, PMID: 9268940

[ref32] Kerstman E. Ahn S. Battu S. Tariq S. Grabois M. (2013). Neuropathic pain. Handb. Clin. Neurol. 110, 175–187. doi: 10.1016/B978-0-444-52901-5.00015-0 23312640

[ref33] Labetoulle M. Baudouin C. Calonge M. Merayo-Lloves J. Boboridis K. G. Akova Y. A.. (2019). Role of corneal nerves in ocular surface homeostasis and disease. Acta Ophthalmol. 97, 137–145. doi: 10.1111/aos.13844, PMID: 30225941

[ref34] Li R. Li D. Wu C. Ye L. Wu Y. Yuan Y.. (2020). Nerve growth factor activates autophagy in Schwann cells to enhance myelin debris clearance and to expedite nerve regeneration. Theranostics 10, 1649–1677. doi: 10.7150/thno.40919, PMID: 32042328 PMC6993217

[ref35] Lobsiger C. S. Cleveland D. W. (2007). Glial cells as intrinsic components of non-cell-autonomous neurodegenerative disease. Nat. Neurosci. 10, 1355–1360. doi: 10.1038/nn1988, PMID: 17965655 PMC3110080

[ref36] Mackie I. Fraunfelder F. Roy F., Current ocular therapy. 4th ed. Philadelphia, PA: WB Saunders (1995): p. 506–508.

[ref37] Mandahl A. (1993). Hypertonic saline test for ophthalmic nerve impairment. Acta Ophthalmol. 71, 556–559. doi: 10.1111/j.1755-3768.1993.tb04636.x, PMID: 8249592

[ref38] Mantelli F. Nardella C. Tiberi E. Sacchetti M. Bruscolini A. Lambiase A. (2015). Congenital corneal Anesthesia and neurotrophic keratitis: diagnosis and management. Biomed. Res. Int. 2015:805876, 1–8. doi: 10.1155/2015/805876, PMID: 26451380 PMC4588028

[ref39] Marfurt C. F. Cox J. Deek S. Dvorscak L. (2010). Anatomy of the human corneal innervation. Exp. Eye Res. 90, 478–492. doi: 10.1016/j.exer.2009.12.010, PMID: 20036654

[ref40] Mastropasqua L. Lanzini M. Dua H. S. D’ Uffizi A. di Nicola M. Calienno R.. (2020). In vivo evaluation of corneal nerves and epithelial healing after treatment with recombinant nerve growth factor for neurotrophic keratopathy. Am J. Ophthalmol. 217, 278–286. doi: 10.1016/j.ajo.2020.04.036, PMID: 32387431

[ref41] Moein H. R. Kheirkhah A. Muller R. T. Cruzat A. C. Pavan-Langston D. Hamrah P. (2018). Corneal nerve regeneration after herpes simplex keratitis: a longitudinal in vivo confocal microscopy study. Ocul. Surf. 16, 218–225. doi: 10.1016/j.jtos.2017.12.001, PMID: 29305292 PMC5889330

[ref42] Morishige N. Komatsubara T. Chikama T. I. Nishida T. (1999). Direct observation of corneal nerve fibres in neurotrophic keratopathy by confocal biomicroscopy. Lancet 354, 1613–1614. doi: 10.1016/S0140-6736(99)04198-7, PMID: 10560682

[ref43] Müller R. T. Abedi F. Cruzat A. Witkin D. Baniasadi N. Cavalcanti B. M.. (2015). Degeneration and regeneration of subbasal corneal nerves after infectious keratitis: a longitudinal in vivo confocal microscopy study. Ophthalmology 122, 2200–2209. doi: 10.1016/j.ophtha.2015.06.047, PMID: 26256833 PMC4623997

[ref44] Müller L. J. Marfurt C. F. Kruse F. Tervo T. M. T. (2003). Corneal nerves: structure, contents and function. Exp. Eye Res. 76, 521–542. doi: 10.1016/S0014-4835(03)00050-2, PMID: 12697417

[ref45] NaPier E. Camacho M. McDevitt T. F. Sweeney A. R. (2022). Neurotrophic keratopathy: current challenges and future prospects. Ann. Med. 54, 666–673. doi: 10.1080/07853890.2022.2045035, PMID: 35243932 PMC8903790

[ref46] Pan Q. Angelina A. Marrone M. Stark W. J. Akpek E. K. (2017). Autologous serum eye drops for dry eye. Cochrane Database Syst. Rev. 2:Cd009327. doi: 10.1002/14651858.CD009327.pub3, PMID: 28245347 PMC5510593

[ref47] Patel S. V. Erie J. C. McLaren J. Bourne W. M. (2007). Keratocyte density and recovery of subbasal nerves after penetrating keratoplasty and in late endothelial failure. Arch. Ophthalmol. 125, 1693–1698. doi: 10.1001/archopht.125.12.1693, PMID: 18071124

[ref48] Pedrotti E. Bonacci E. Chierego C. de Gregorio A. Cozzini T. Brighenti T.. (2022). Eight months follow-up of corneal nerves and sensitivity after treatment with cenegermin for neurotrophic keratopathy. Orphanet J. Rare Dis. 17:63. doi: 10.1186/s13023-022-02237-5, PMID: 35189948 PMC8862484

[ref49] Pflugfelder S. C. Massaro-Giordano M. Perez V. L. Hamrah P. Deng S. X. Espandar L.. (2020). Topical recombinant human nerve growth factor (Cenegermin) for neurotrophic keratopathy: a multicenter randomized vehicle-controlled pivotal trial. Ophthalmology 127, 14–26. doi: 10.1016/j.ophtha.2019.08.020, PMID: 31585826

[ref50] Roszkowska A. M. Inferrera L. Aragona E. Gargano R. Postorino E. I. Aragona P. (2022). Clinical and instrumental assessment of the corneal healing in moderate and severe neurotrophic keratopathy treated with rh-NGF (Cenegermin). Eur. J. Ophthalmol. 32, 3402–3410. doi: 10.1177/11206721221097584, PMID: 35473440

[ref51] Sacchetti M. Lambiase A. (2014). Diagnosis and management of neurotrophic keratitis. Clin. Ophthalmol. 8, 571–579. doi: 10.2147/OPTH.S45921, PMID: 24672223 PMC3964170

[ref52] Smeyne R. J. Klein R. Schnapp A. Long L. K. Bryant S. Lewin A.. (1994). Severe sensory and sympathetic neuropathies in mice carrying a disrupted Trk/NGF receptor gene. Nature 368, 246–249. doi: 10.1038/368246a0, PMID: 8145823

[ref53] Tepelus T. C. Chiu G. B. Huang J. Huang P. Sadda S. V. R. Irvine J.. (2017). Correlation between corneal innervation and inflammation evaluated with confocal microscopy and symptomatology in patients with dry eye syndromes: a preliminary study. Graefes Arch. Clin. Exp. Ophthalmol. 255, 1771–1778. doi: 10.1007/s00417-017-3680-3, PMID: 28528377

[ref54] Versura P. Giannaccare G. Pellegrini M. Sebastiani S. Campos E. C. (2018). Neurotrophic keratitis: current challenges and future prospects. Eye Brain 10, 37–45. doi: 10.2147/EB.S117261, PMID: 29988739 PMC6029608

[ref55] Xie C. Liu B. Zhao X. He Q. Liu L. Wei R. (2022). Characteristics of the ocular surface in neurotrophic keratitis induced by trigeminal nerve injury following neurosurgery. Int. Ophthalmol. 43, 1229–1240. doi: 10.1007/s10792-022-02521-0, PMID: 36115903 PMC10113358

[ref56] Yavuz Saricay L. Bayraktutar B. N. Lilley J. Mah F. S. Massaro-Giordano M. Hamrah P. (2022). Efficacy of recombinant human nerve growth factor in stage 1 neurotrophic keratopathy. Ophthalmology 129, 1448–1450. doi: 10.1016/j.ophtha.2022.08.014, PMID: 35973594

[ref57] You L. Kruse F. E. Völcker H. E. (2000). Neurotrophic factors in the human cornea. Invest. Ophthalmol. Vis. Sci. 41, 692–702. PMID: 10711683

