# Supplementary material for: Effect of recombinant human nerve growth factor treatment on corneal nerve regeneration in patients with neurotrophic keratopathy
Source: Front Neurosci. 2023 Oct 30;17:1210179. doi: 10.3389/fnins.2023.1210179 (PMC10642242; doi:10.3389/fnins.2023.1210179)
Supplement: Supplementary file 1 [file Table_1.DOCX]

Supplementary Tables

**Supplementary Table 1.**

Concurrent treatments

| **Concurrent Treatments** | Subjects, n (%) |
| --- | --- |
| Loteprednol Etabonate Ophthalmic Suspension 0.5% | 32.0% |
| Autologous Serum Tears | 20.0% |
| Moxifloxacin Ophthalmic Solution 0.5% | 8.0% |
| Artificial Tears | 8.0% |
| Cyclosporine Ophthalmic Emulsion 0.05% | 4.0% |
| Lifitegrast Ophthalmic Solution 5% | 4.0% |

**Supplementary Table 2.**

Prevalence of symptoms pre- and post-rhNGF treatment.

| **Symptom** | **Pre-treatment, n (%)** | **Post-treatment,**  **n (%)** |
| --- | --- | --- |
| Blurry Visual Acuity | 11 (44.0%) | 5 (20.0%) |
| Sensitivity to Light | 10 (40.0%) | 10 (40.0%) |
| Irritation | 8 (32.0%) | 8 (32.0%) |
| Foreign Body Sensation | 6 (24.0%) | 4 (16.0%) |
| Burning | 5 (20.0%) | 4 (16.0%) |
| Itchiness | 4 (16.0%) | 3 (12.0%) |
| Pain | 3 (12.0%) | 0 (0%) |
| Floaters | 3 (12.0%) | 1 (4.0%) |
| Redness | 3 (12.0%) | 0 (0%) |
| Discomfort | 2 (8.0%) | 1 (4.0%) |
| Diplopia | 2 (8.0%) | 0 (0%) |
| Dryness | 1 (4.0%) | 1 (4.0%) |
| Tearing | 1 (4.0%) | 0 (0%) |
| Flashes | 1 (4.0%) | 0 (0%) |
